# Supplementary material for: Fibre Bragg Grating Based Interface Pressure Sensor for Compression Therapy
Source: Sensors (Basel). 2022 Feb 24;22(5):1798. doi: 10.3390/s22051798 (PMC8915074; doi:10.3390/s22051798)
Supplement: Supplementary file 1 [file sensors-22-01798-s001.zip › sensors-1577295-supplementary.pdf]

sensors

MDPI

Article

Supplementary Data: Fibre Bragg Grating Based Interface Pressure Sensor for Compression Therapy

James A. Bradbury [1,†], Qimei Zhang [2,†], Francisco U. Hernandez Ledezma [3,†], Ricardo Correia [1], Serhiy Korposh [1], Barrie R. Hayes-Gill [1], Ferdinand Tamoué [4], Alison Parnham [5], Simon A. McMaster [3] and Stephen P. Morgan [1,3,*]

1 Optics and Photonics Group, Faculty of Engineering, University of Nottingham, Nottingham NG7 2RD, UK; james.bradbury@nottingham.ac.uk (J.A.B.); ricardo.goncalvescorreia@nottingham.ac.uk (R.C.); s.korposh@nottingham.ac.uk (S.K.); barrie.hayes-gill@nottingham.ac.uk (B.R.H.-G.)
2 Department of Engineering, School of Science and Technology, Nottingham Trent University Nottingham, Nottingham NG1 4FQ, UK; qimei.zhang@ntu.ac.uk
3 Footfalls and Heartbeats (UK) Limited, 10 Castle Quay, Castle Boulevard, Nottingham NG7 1FW, UK; ulises@footfallsandheartbeats.com (F.U.H.L.), simon@footfallsandheartbeats.com (S.A.M.)
4 KOB GmbH, Lauterstraße 50, 67752 Wolfstein, Germany; ferdinand.tamoue@kob.de
5 School of Health Sciences, University of Nottingham, Nottingham NG7 2RD, UK; alison.parnham@nottingham.ac.uk
* Correspondence: steve.morgan@nottingham.ac.uk
† These authors contributed equally to this work.

Citation: Bradbury, J.A.; Zhang, Q.; Hernandez Ledezma, F.U.; Correia, R.; Korposh, S.; Hayes-Gill, B.R.; Tamoué, F.; Parnham, A.; McMaster, S.A.; Morgan, S.P. Fibre Bragg Grating Based Interface Pressure Sensor for Compression Therapy. *Sensors* **2022**, *22*, 1798. https://doi.org/10.3390/s22051798

Academic Editors: Thomas Geernaert, Jeroen Missinne and Geert Van Steenberge

Received: 14 January 2022
Accepted: 22 February 2022
Published: 24 February 2022

Publisher's Note: MDPI stays neutral with regard to jurisdictional claims in published maps and institutional affiliations.

Copyright: © 2022 by the authors. Licensee MDPI, Basel, Switzerland. This article is an open access article distributed under the terms and conditions of the Creative Commons Attribution (CC BY) license (https://creativecommons.org/licenses/by/4.0/).

Figures S1 – S9

Calibration data showing wavelength shift against mass for the ten sensors. This was done using the method described in section 2.3.1 of the main paper.

Figures S10 – S16

Graphs showing the response of the sensor using a blood pressure cuff as described in section 2.3.3 of the main paper.

Figures S17 – S20

Graphs showing the response of the sensor compared to the response of the gold standard sensor when used under a compression bandage, using the method as described in section 2.4.2 of the main paper.
Note that in figures S19 and S20 a two layer bandage system was used.

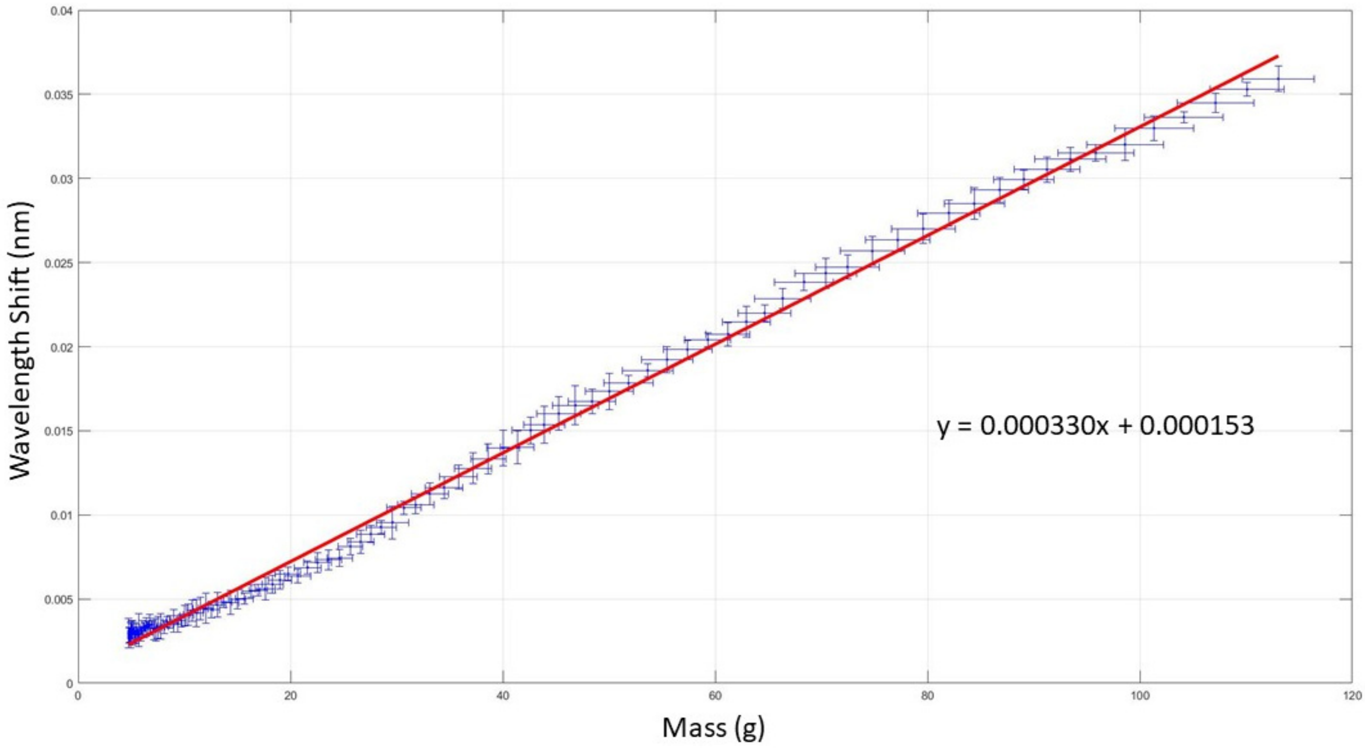

Figure S1 Calibration Data 2

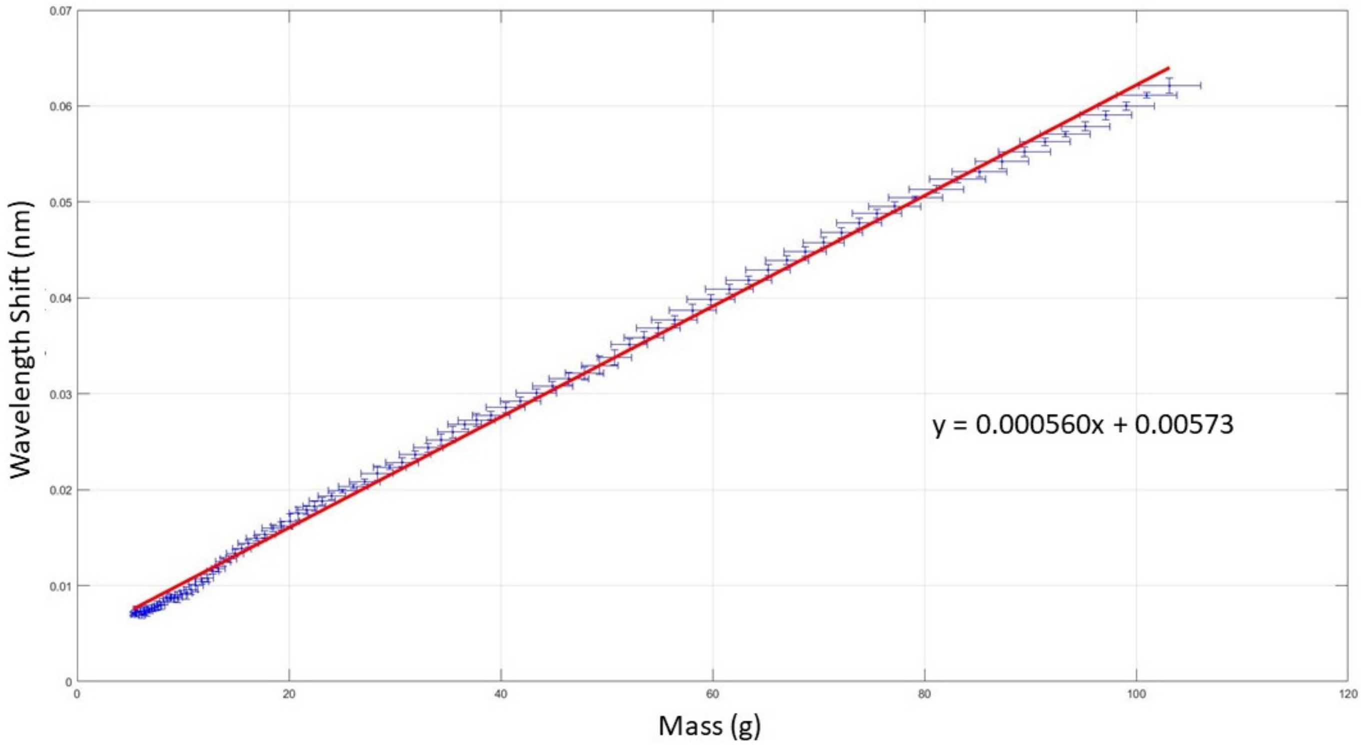

Figure S2 Calibration Data 3

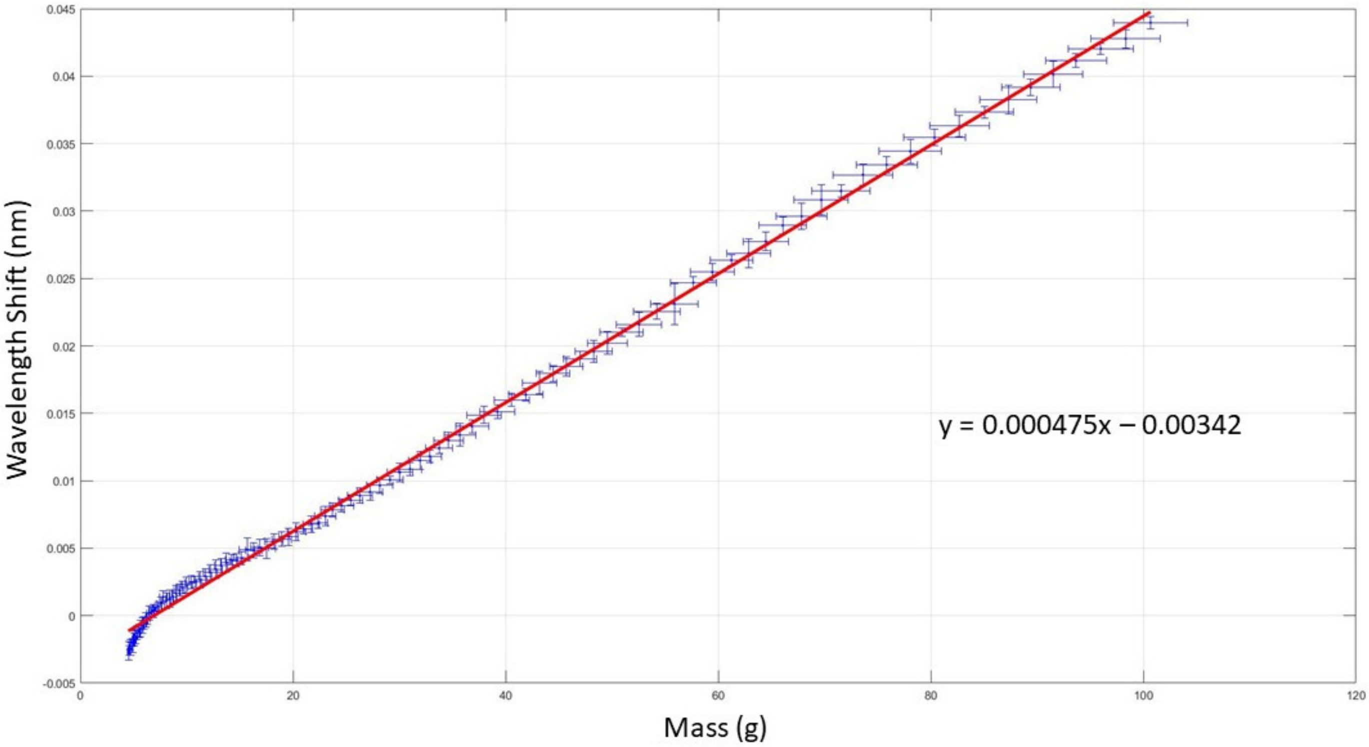

Figure S3 Calibration Data 4

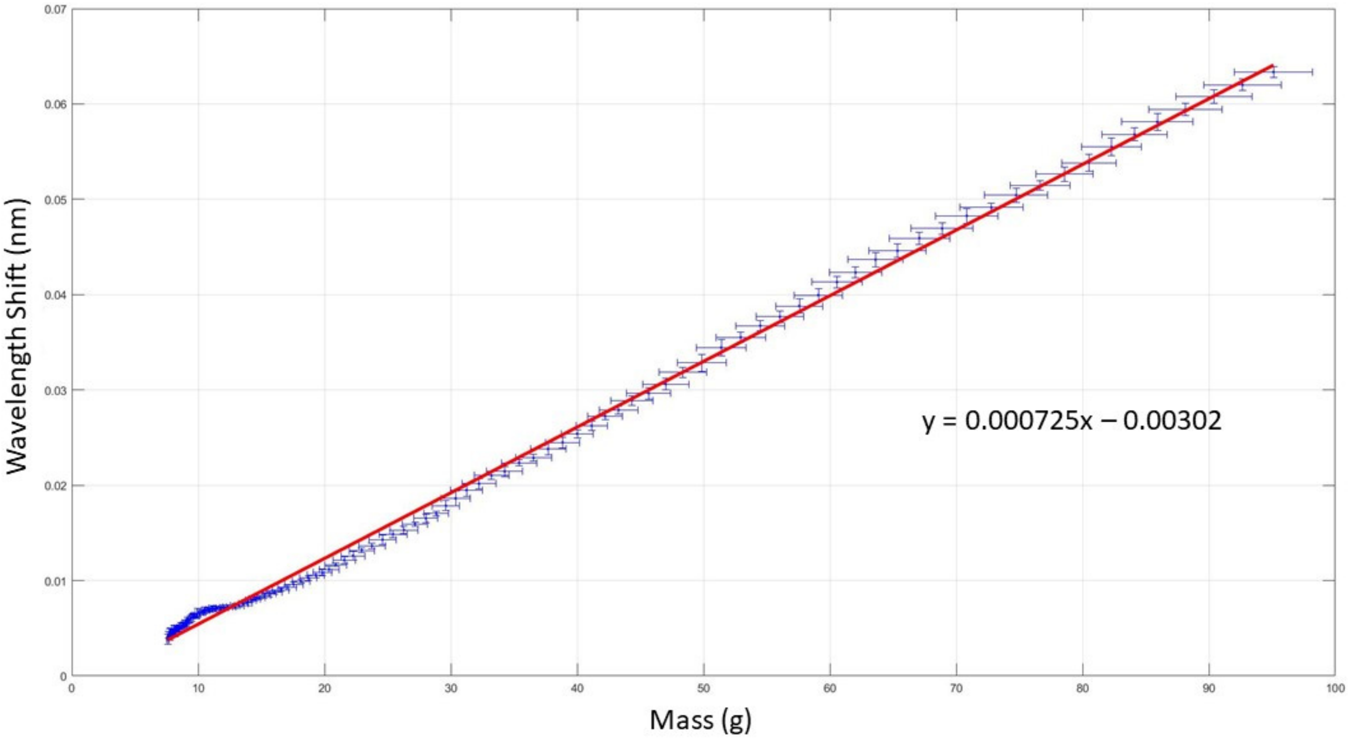

Figure S4 Calibration Data 5

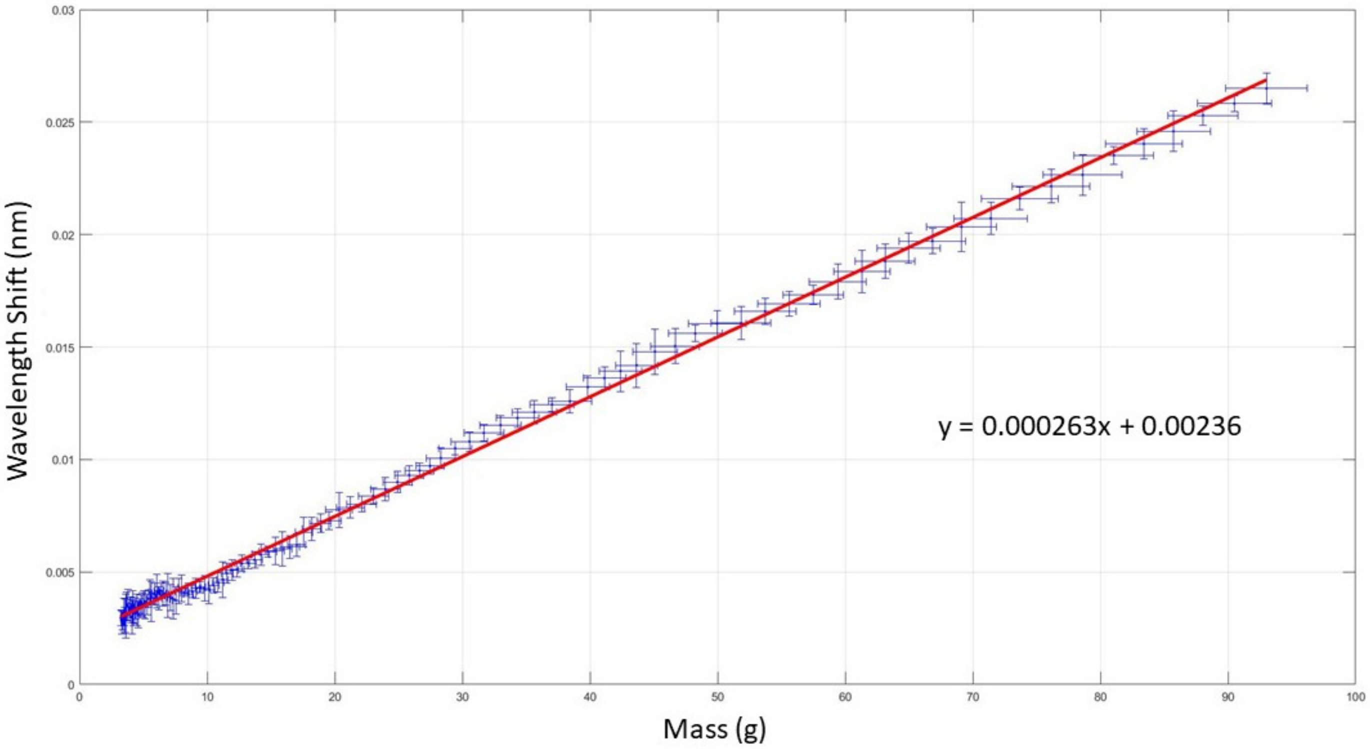

Figure S5 Calibration Data 6

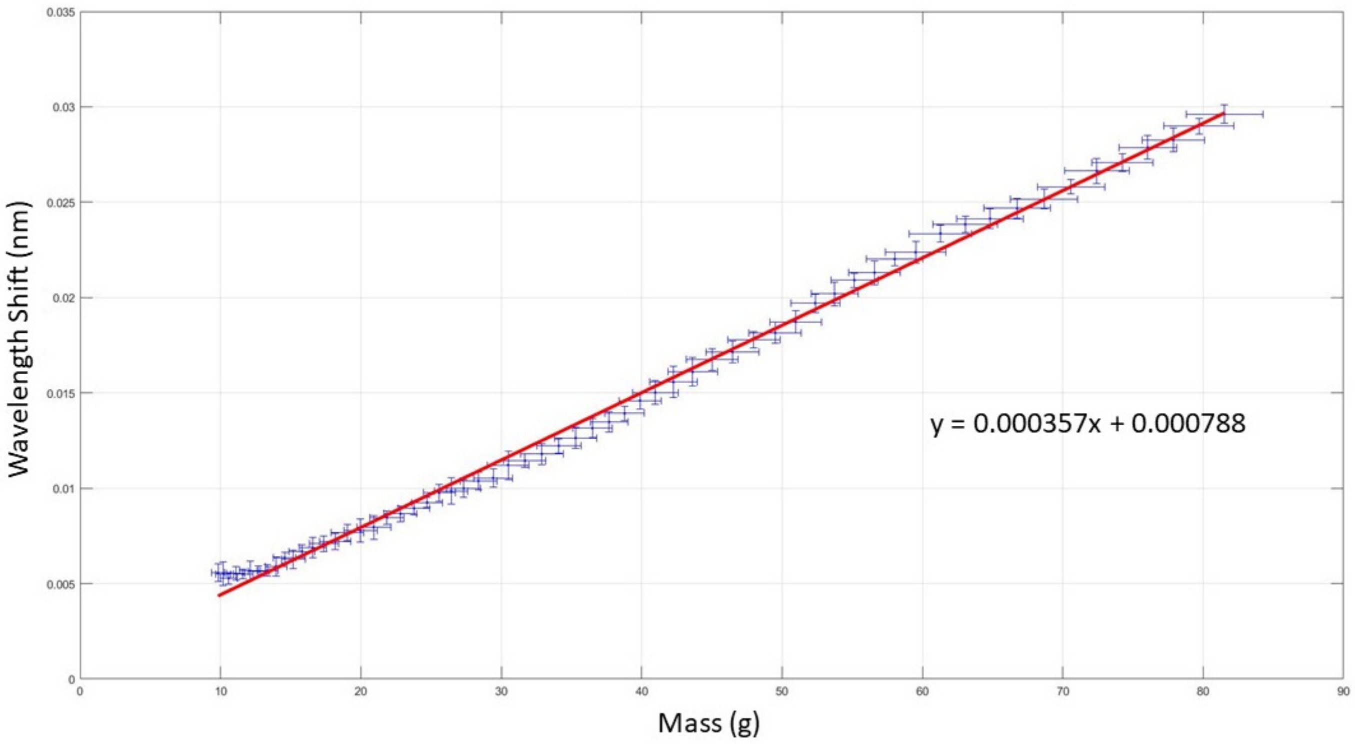

Figure S6 Calibration Data 7

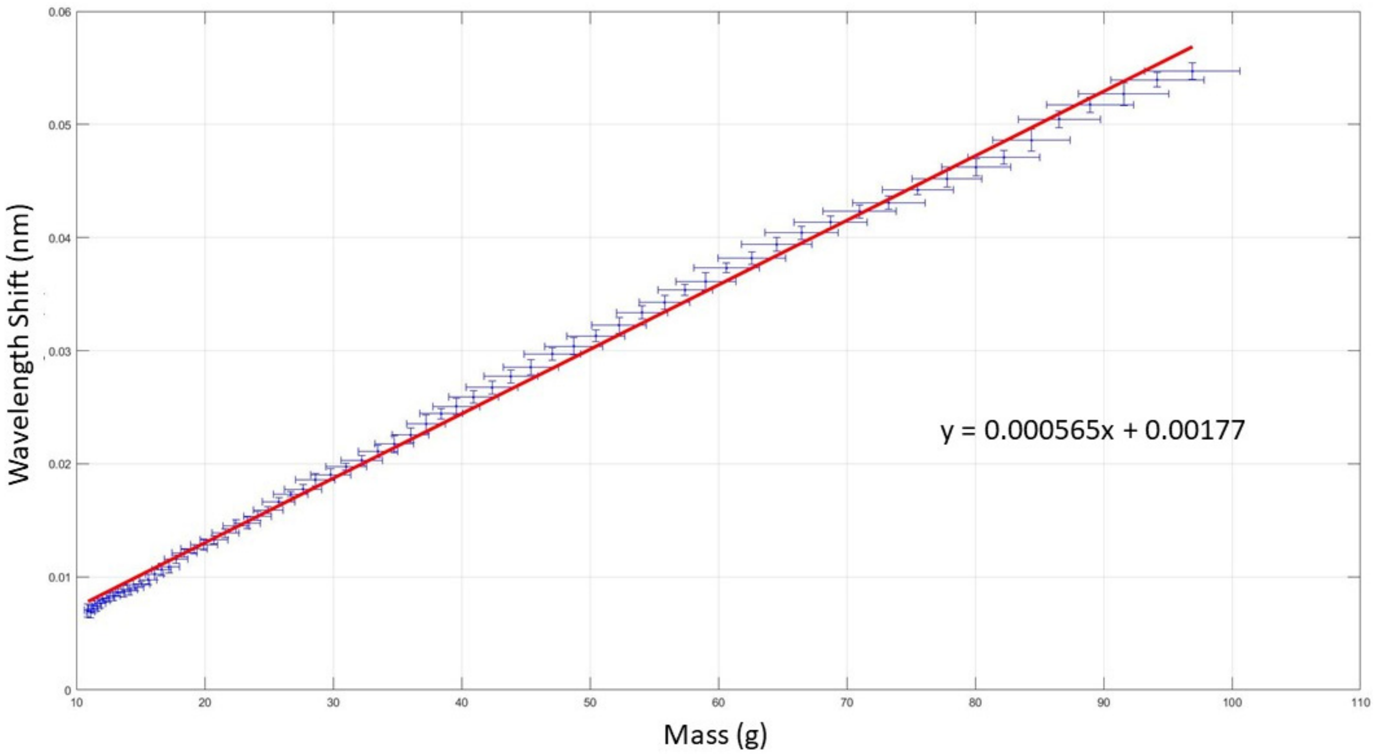

Figure S7 Calibration Data 8

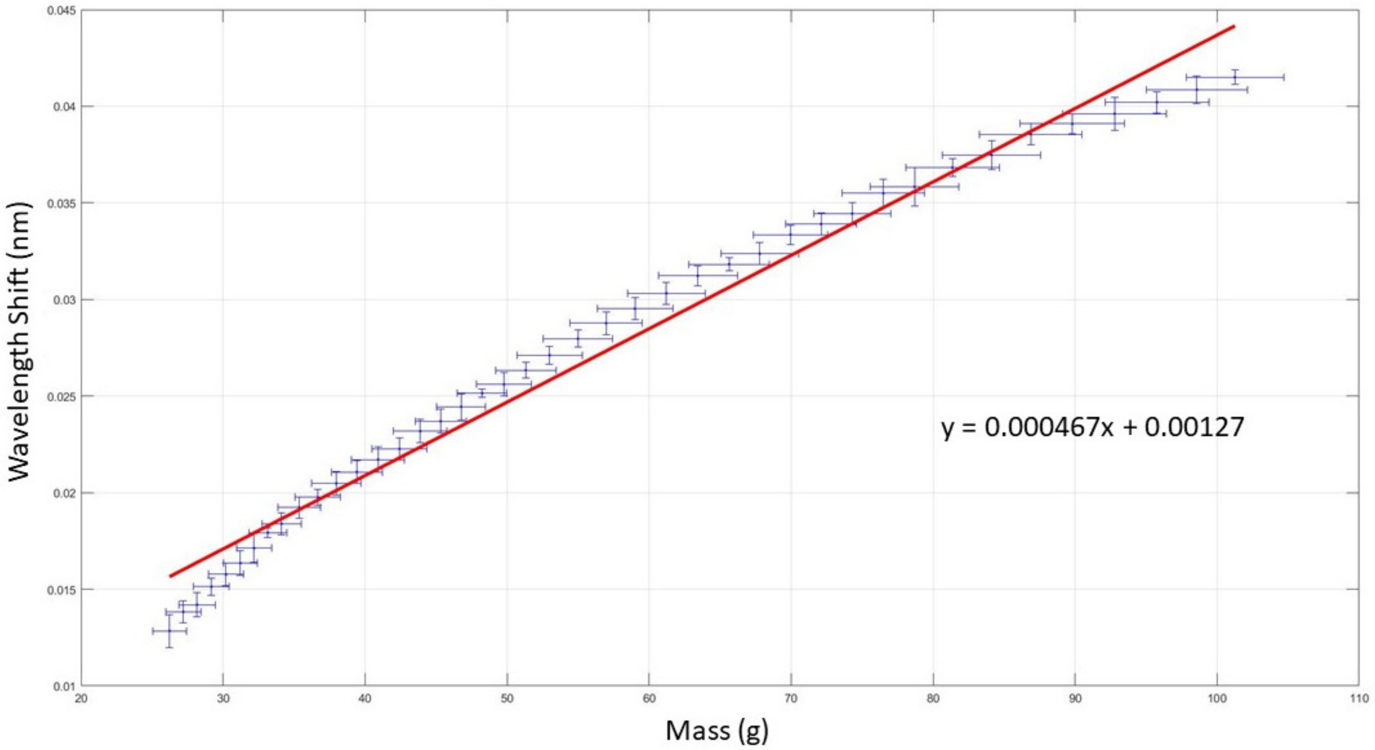

Figure S8 Calibration Data 9

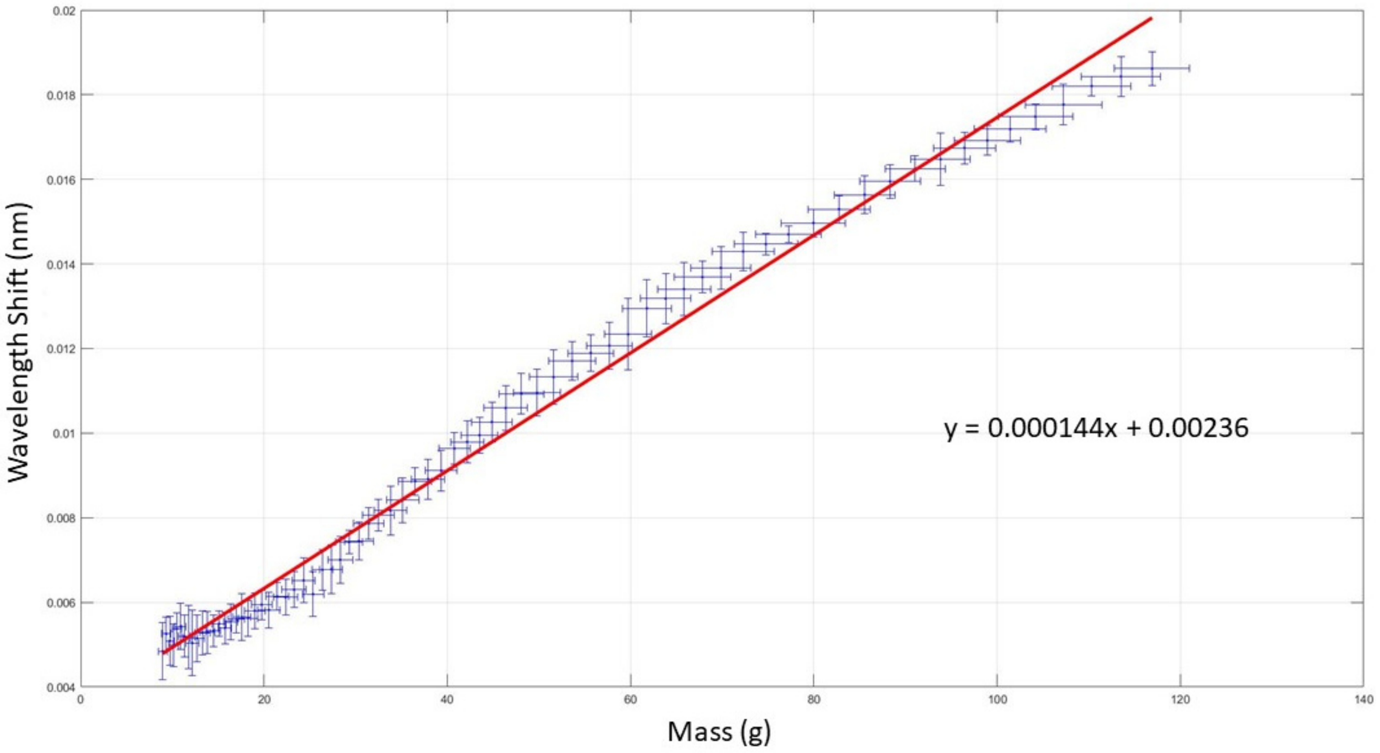

Figure S9 Calibration Data 10

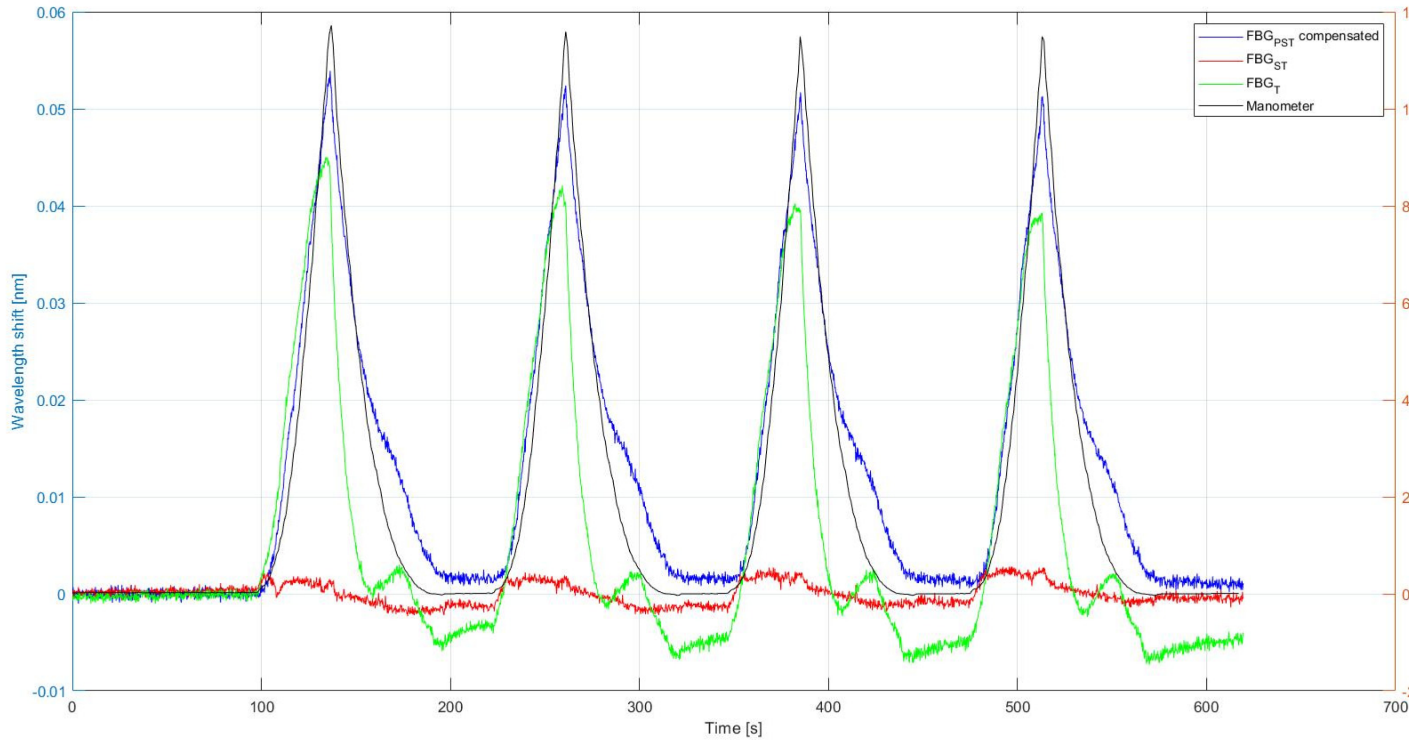

Figure S20 Pressure Cuff Data 2

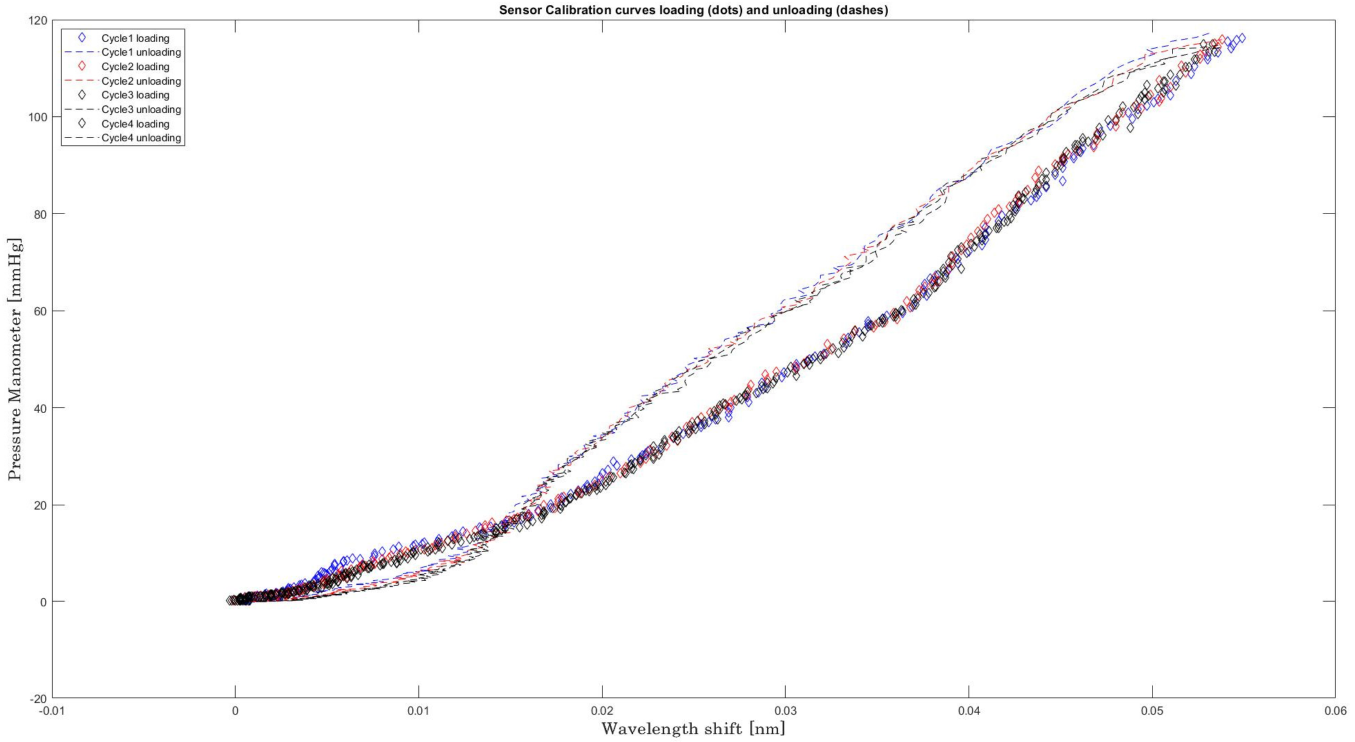

Figure S31 Pressure Cuff Load and Unload Data 2

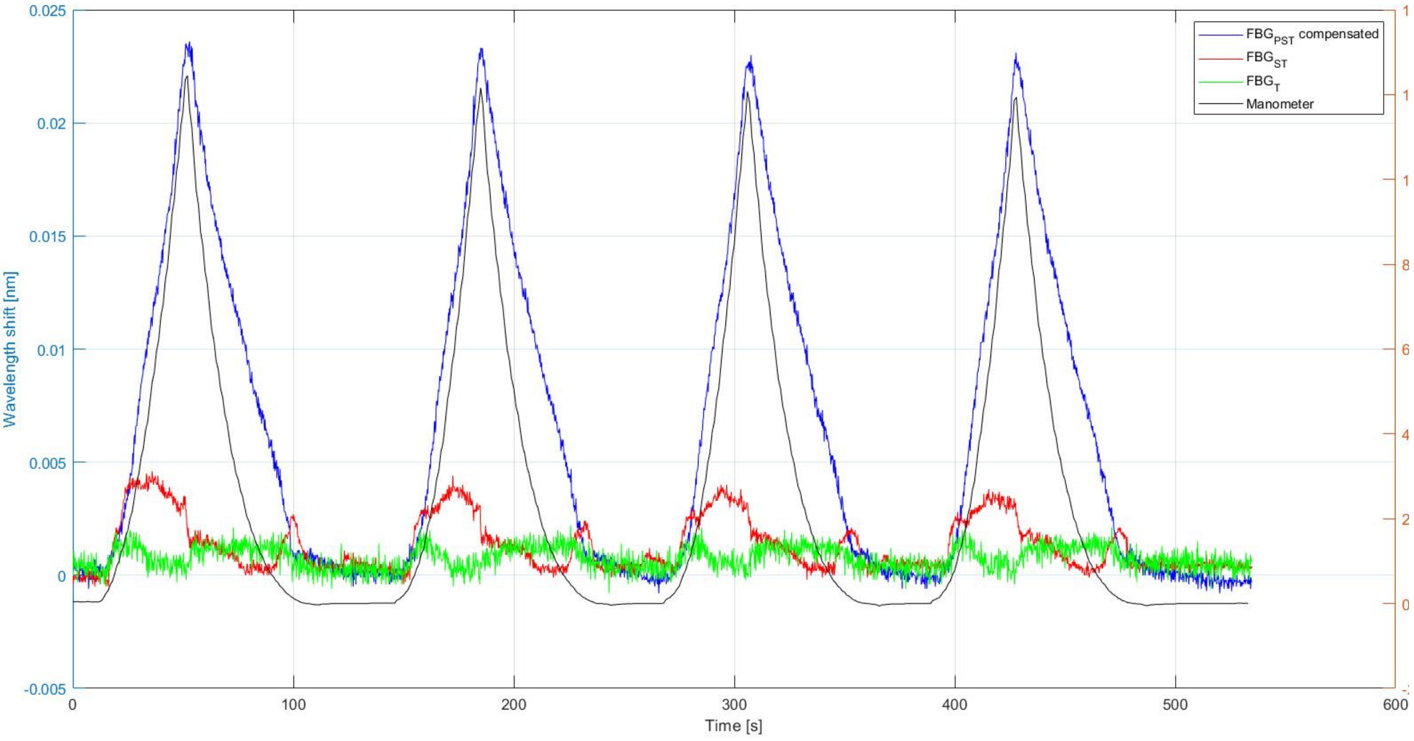

Figure S42 Pressure Cuff Data 3

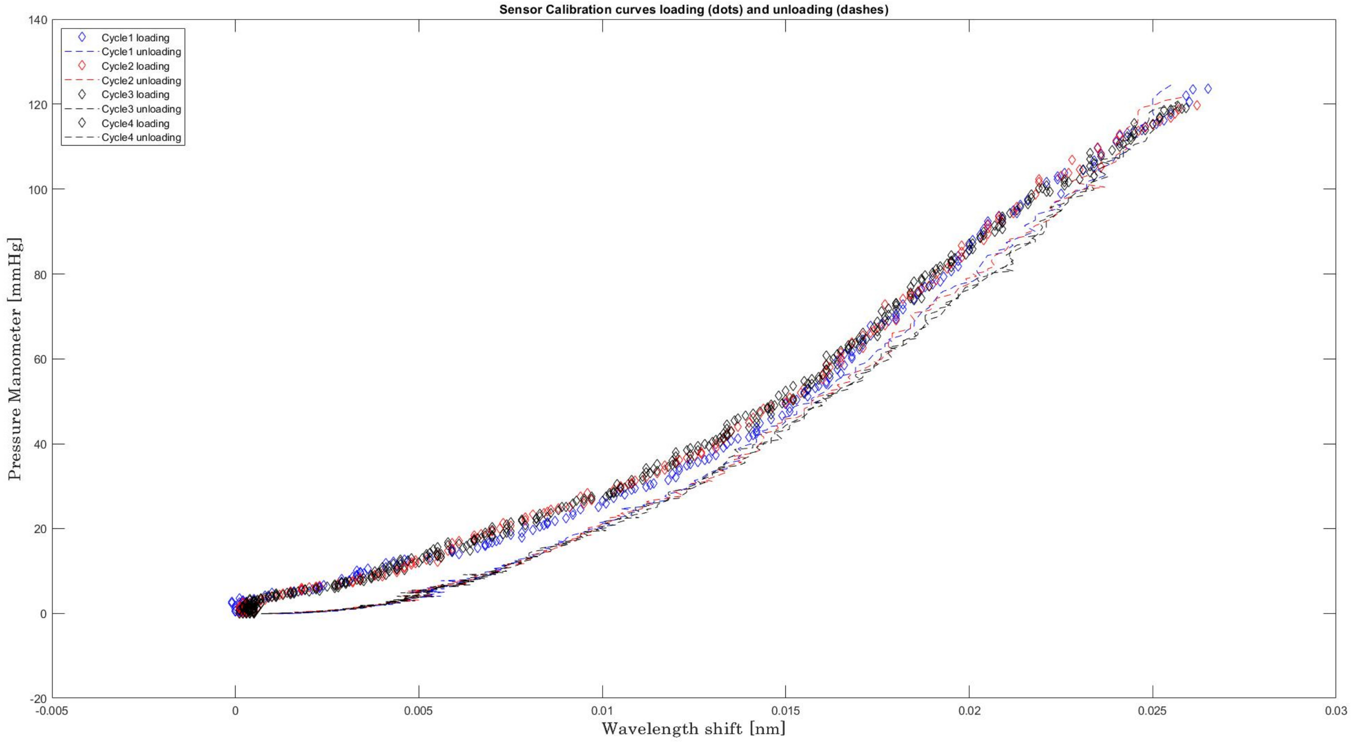

Figure S53 Pressure Cuff Load and Unload Data 3

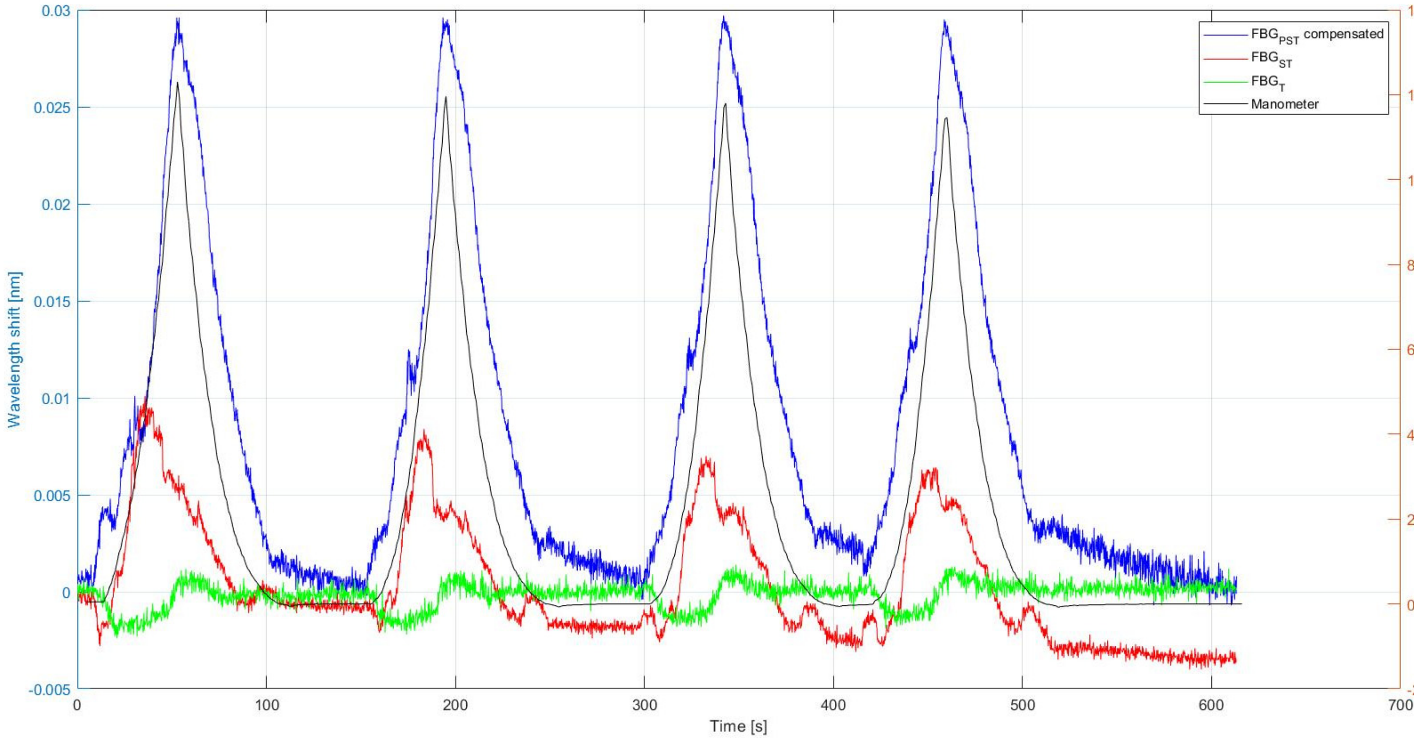

Figure S64 Pressure Cuff Data 4

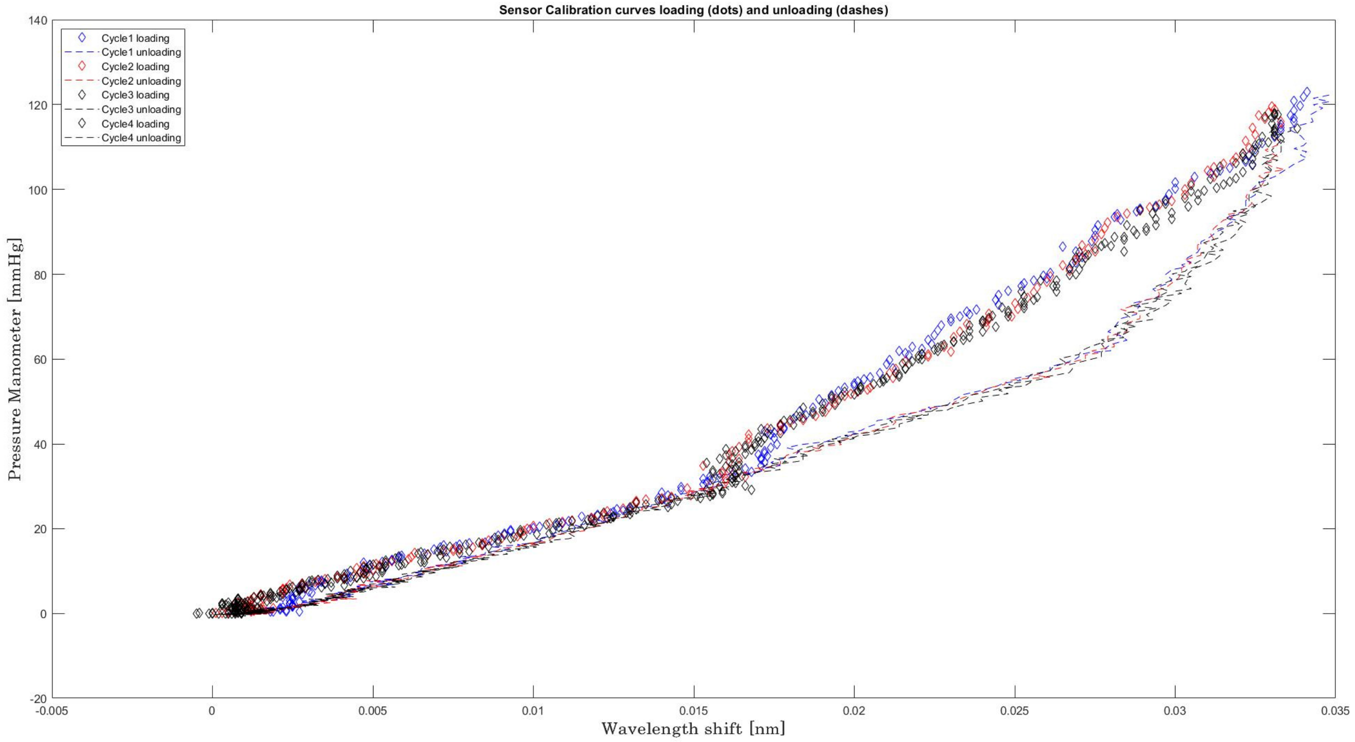

Figure S75 Pressure Cuff Load and Unload Data 4

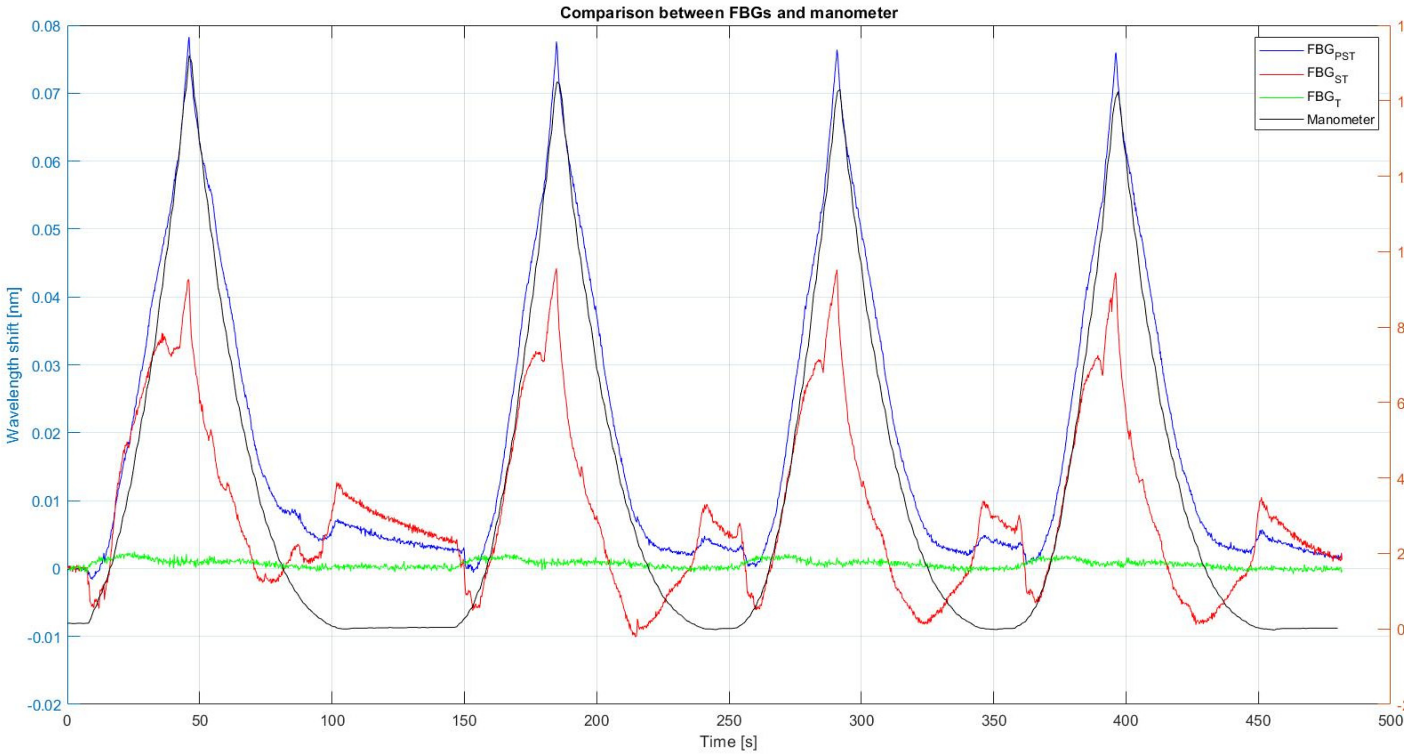

Figure S86 Pressure Cuff Data 5

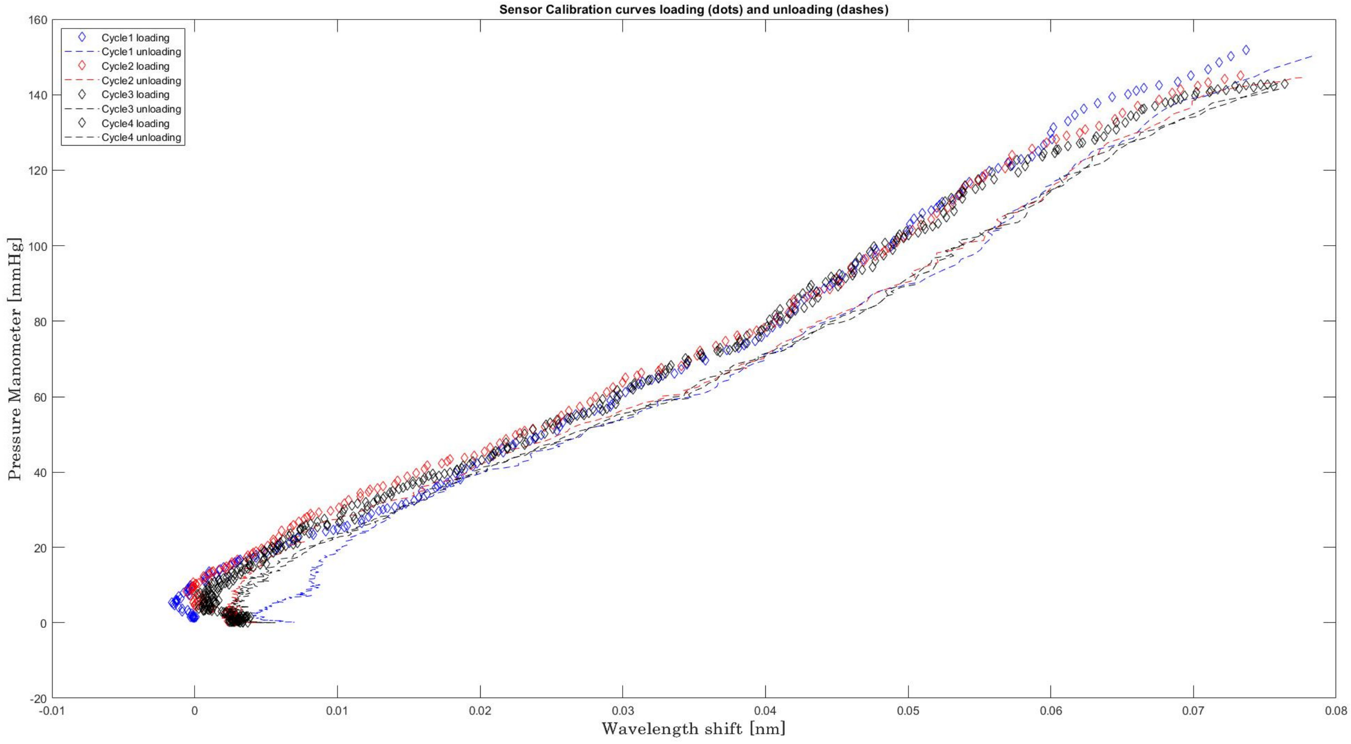

Figure S97 Pressure Cuff Load and Unload Data 5

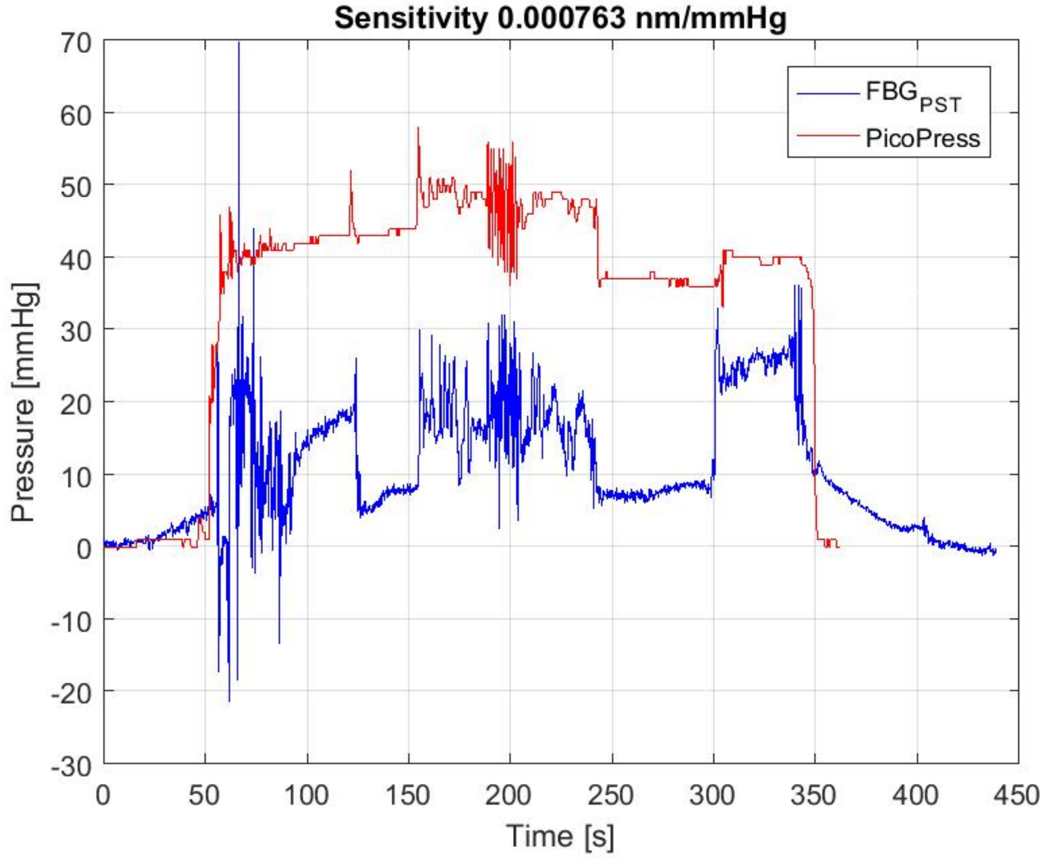

Figure S108 Wrapping Data 2

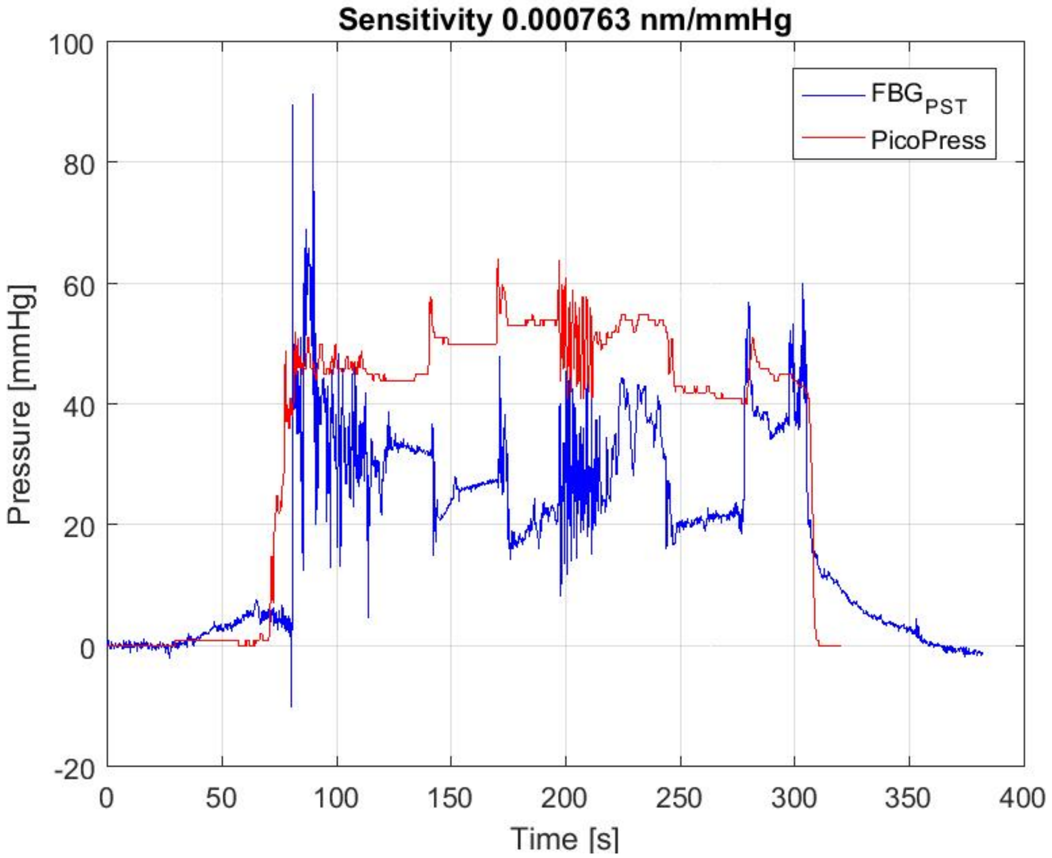

Figure S119 Wrapping Data 3

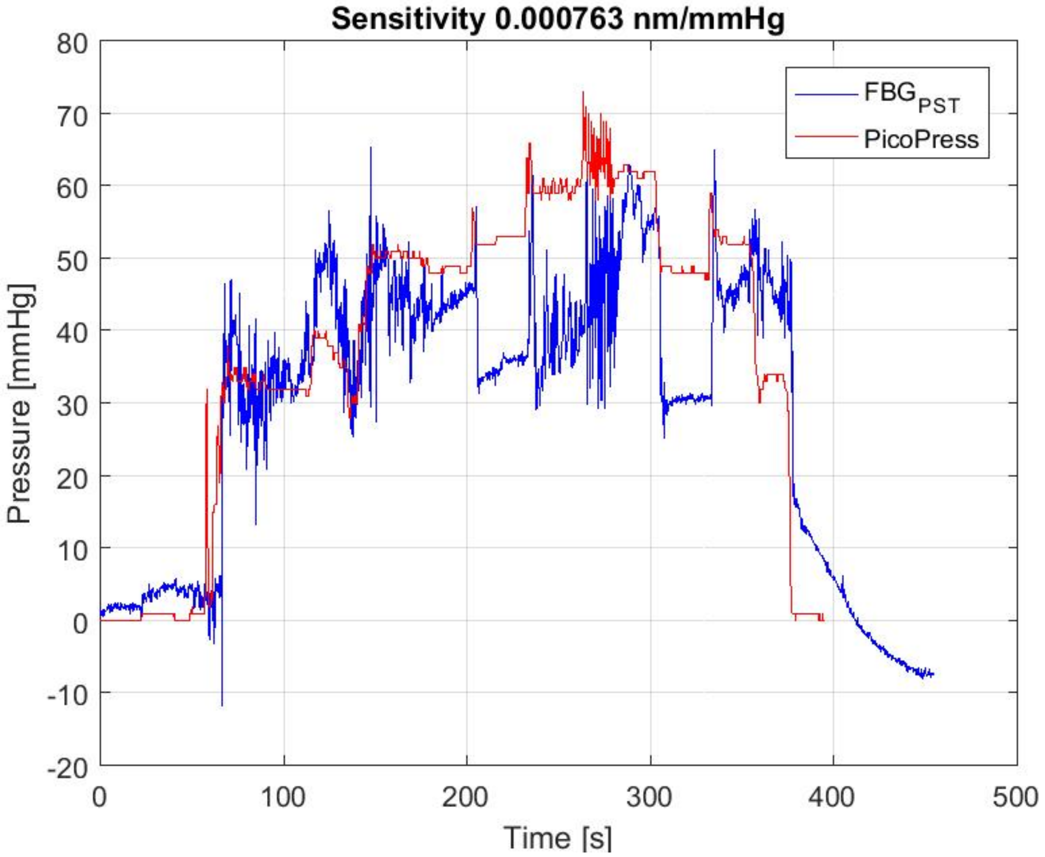

Figure S20 Wrapping Data 4

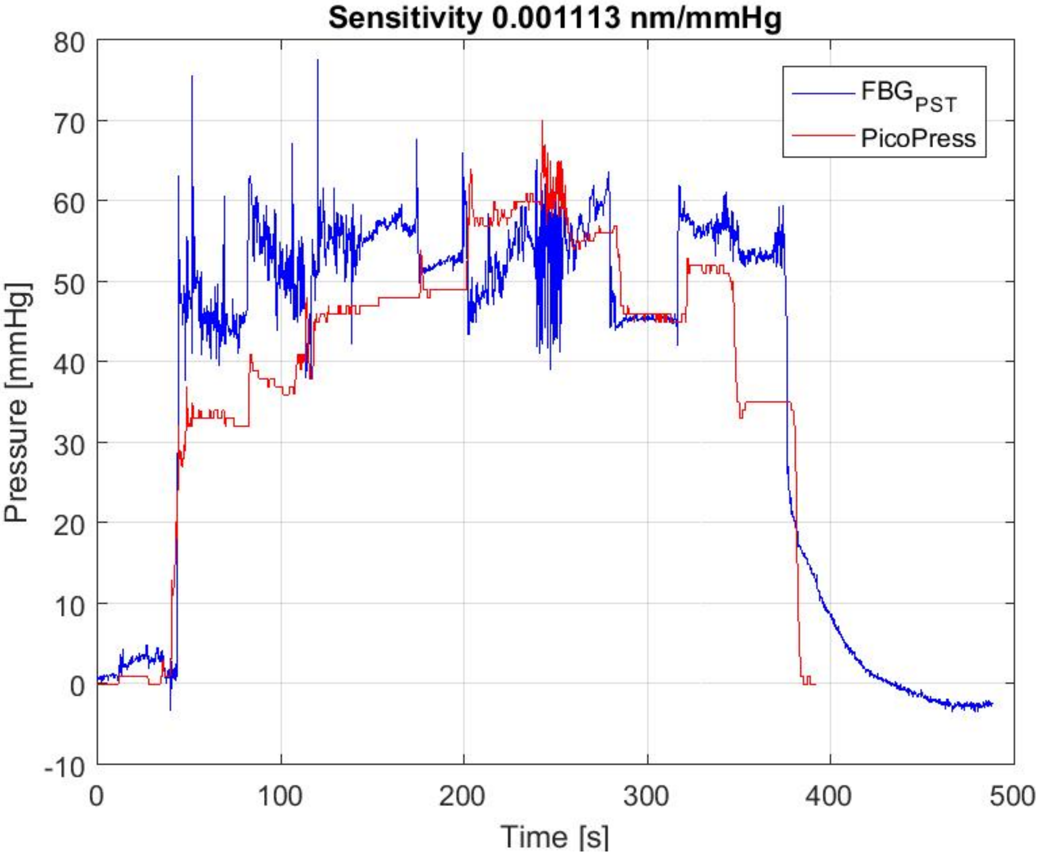

Figure S21 Wrapping Data 5

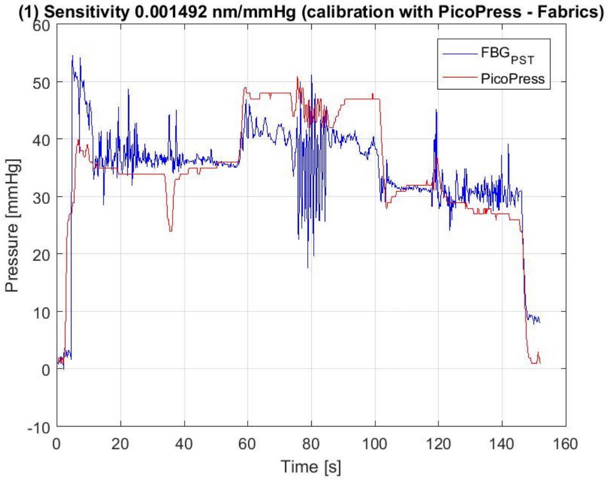

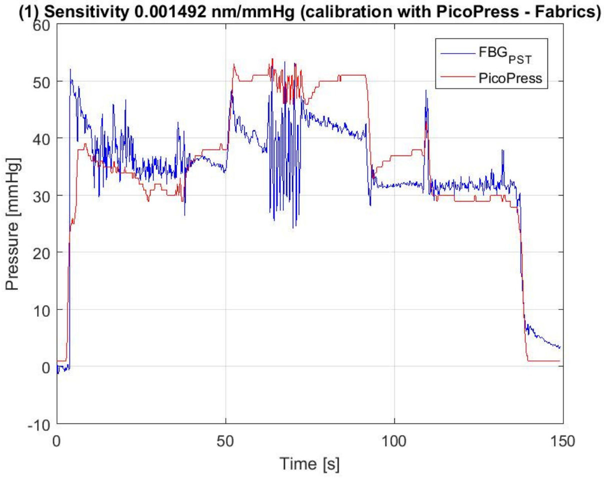

Figure S22 Wrapping Data repeated with the same sensor
